# Lived experiences of maternal near misses: a qualitative study in the Kilimanjaro Region, Tanzania

**DOI:** 10.1186/s12978-025-02018-w

**Published:** 2025-05-26

**Authors:** Enna G. Sengoka, Gunilla Björling, Michael J. Mahande, Janet Mattsson, Gileard Masenga

**Affiliations:** 1 School of Health and Welfare, Jönköping University, Jönköping, Sweden; 2 Faculty of Nursing, Kilimanjaro Christian Medical Centre (KCMC) University, Moshi, Tanzania; 3 Department of Obstetrics and Gynaecology, Kilimanjaro Christian Medical Centre, Moshi, Tanzania; 4 Department of Neurobiology, Care Sciences, and Society, Karolinska Institutet, Stockholm, Sweden; 5 Faculty of Public Health, KCMC University, Moshi, Tanzania; 6 Faculty of Health and Social Sciences, University of Southeast Norway, Borre, Norway; 7 Department of Health and Caring Sciences, University of Linnaeus, Växjö, Sweden; 8 Faculty of Medicine, KCMC University, Moshi, Tanzania

**Keywords:** Maternal near-miss, Severe maternal morbidity, Near-miss women, Lived experience, Qualitative study, Tanzania

## Abstract

**Background:**

In recent years, Tanzania has significantly reduced maternal mortality rates; however, pregnant women in Tanzania still face severe health risks and complications. The rate of maternal near misses is nearly 60% higher in Tanzania compared to other low-income countries. Women who survive severe complications during pregnancy or childbirth may experience long-lasting adverse effects, such as poor physical health, impaired sexual function, psychological distress, and a decreased quality of life. Therefore, our research aimed to understand the lived experiences of Tanzanian women who survived severe maternal complications within their cultural and social context.

**Method:**

By using the Sub-Saharan Africa criteria for near misses, twelve (12) women who survived severe maternal complications were recruited between August and September 2022. The study utilized a descriptive qualitative design with an inductive approach to explore women's lived experiences of a maternal near miss. The participants were purposively sampled and interviewed face-to-face in the hospital or their homes six weeks after discharge from the hospital. Recorded interviews were transcribed and analysed using Graneheim and Lundman's content analysis.

**Results:**

The analysis revealed four themes and eight subthemes. The themes were *living with severe maternal complications, impact on social life, perception of obstetric care services* and *person-centred care*. The informants described pain experiences, weakness, fear, financial difficulties, neglect, long waiting times, and a lack of information. However, they also mentioned a need for increased awareness of danger signs, care satisfaction, and the importance of close relatives' social support.

**Conclusion:**

Maternal near misses have a great impact on women's physical, financial, and mental well-being. Women also experience long service waiting times, communication barriers, and neglect. Good healthcare, person-centred care, patient education, and psychological support can improve women’s lived experiences.

## Abstract (highlights)

This study delves into the personal experiences of women who have encountered life-threatening complications during pregnancy, childbirth, or postpartum. The research was conducted in Kilimanjaro, Tanzania, in 2022 at both the zonal and district referral hospitals. The study included twelve women aged between 21 and 41 who shared their narratives, revealing how their struggles with maternal complications had negatively affected their physical, mental, and financial well-being. They noted that extended waiting times, communication barriers, and neglect were perceived as indicators of poor-quality obstetric care services. However, the study also highlighted that close family members' support played a significant role in the mothers' recovery. According to the study, providing person-centred care and patient education can be useful in this group, as mothers who experience near-miss situations are more aware of warning signs and satisfied with the care they receive.

## Introduction

Pregnancy and giving birth are natural processes that most women experience without complications. Nevertheless, there are situations where the health risks can be severe, and the most severe outcome could be the mother's death related to complications [1]. Severe complications during pregnancy and childbirth resulted in the loss of approximately 287,000 women's lives globally, which means that almost 800 maternal deaths occur daily. Out of these deaths, Sub-Saharan Africa and Southern Asia account for approximately 87%; Sub-Saharan Africa alone accounts for approximately 70% (202,000), and Southern Asia accounts for 16% (47,000) [2, 3], while Tanzania accounts for 238,000 [4]. Over the last 20 years, there has been growing attention given to maternal near misses in the field of maternal health due to the decrease in maternal deaths and the extremely low maternal mortality ratio in developed nations, which has prompted the exploration of cases of maternal near misses [5]. As maternal health complications occur more frequently than maternal deaths, maternal near miss has been identified as a more useful metric for assessing maternal health services than maternal death. Additionally, studying maternal near-miss cases is less frightening for healthcare providers because the mothers survived [5].

A maternal near miss (MNM) occurs when a woman experiences a severe complication during pregnancy, childbirth, or within 42 days of giving birth and almost dies but ultimately survives [6, 7]. These incidents occur at different rates around the world. A study of 49 countries found that the occurrence ranges from 3.10 to 31.88 per 1,000 live births, with higher rates in low-income countries than in high-income countries [8], while Tanzania's rate is exceptionally high at 50 per 1,000 live births [9]. The inclusion of patients'opinions in maternal health research has led to a better comprehension of the personal, communal, and societal impact of maternal illness, which is linked with negative consequences such as poor physical health, impaired sexual function, decreased quality of life, and psychological distress. Additionally, severe obstetric complications can disrupt a household's financial stability and a woman's social status. While these findings apply universally, differences are observed in diverse cultures and environments [10]. Various studies have been conducted on maternal near misses, which were mostly descriptive, retrospective, or cross-sectional and relied on secondary data sources such as patient records and files. However, to better understand the care that pregnant women receive, it is essential to consider patients'experiences directly. By analysing the experiences of those who have experienced a maternal near miss, the causes can be identified and compared to create a report that can improve the quality of care in healthcare centres, which can be achieved through explorative studies [11]. In Tanzania, the maternal near-miss rate is alarmingly high [9]. Failing to consider the experiences of these women could result in more mothers suffering from maternal complications. Given the complexity of the physical and psychological challenges they face and the scarcity of studies on this topic in Tanzania, our research aimed to understand the lived experiences of Tanzanian women who survived severe maternal complications within their cultural and social context.

## Methods

### Study design

The study utilized a descriptive qualitative design with an inductive approach using semi-structured in-depth interviews to allow participants to share their views to understand the lived experiences of women who have had a maternal near miss. According to Polit and Beck [12], the opted method was deemed suitable as a qualitative inductive explorative approach that captures the real-life experiences of individuals in a specific context. This study follows Polit and Beck's methods [12] for writing and analysing qualitative research and reporting adhered to the Standards for Reporting Qualitative Research (SRQR) checklist used to report qualitative data [13].

### Researchers’ characteristics

The Principal Investigator (EGS) is a doctoral candidate with specialized knowledge in the field of midwifery, while the research assistant (CJM) is an experienced nurse-midwife with expertise in conducting qualitative research. Both have been trained in interview guide development and research ethics. The interviewers’ background, their reasons for conducting the study, and information about the project were stated at the beginning of all interviews.

### Study setting

The study was conducted at two hospitals: one teaching hospital, Kilimanjaro Christian Medical Centre, a Zonal Referral Consultant Hospital owned by the (ELCT) under the Good Samaritan Foundation of Tanzania (GSF), which is situated in the foothills of Mount Kilimanjaro, approximately 4 km from Moshi municipal, and one district hospital, Hai district hospital, which is located 47 km from Moshi municipal and owned by the government. The selection of these hospitals was based on their provision of Comprehensive Emergency Obstetric and Newborn Care services and their possession of specialized healthcare professionals, such as nurses-midwives and doctors, who can manage obstetric conditions that pose a threat to women's lives.

### Sampling and sampling technique

Upon hospital discharge or hospital stay, a nurse-midwife used purposive sampling to select women who experienced maternal near misses where their addresses and phone numbers of selected informants were obtained. Inclusion criteria to qualify as a participant in the study: the woman needed to be between 15 and 49 years of age, reside within the Kilimanjaro region, speak Kiswahili fluently, and have experienced maternal near misses. Out of the 40 identified women, 20 met these inclusion criteria. Six weeks postdelivery, the principal investigator (ES) contacted each of the 20 potential participants via phone to explain the study's purpose and obtain their consent for a face-to-face interview, which was audio recorded. Of the 20 contacted, 15 verbally consented to participate in the study. The interviews were scheduled conveniently for each woman in both time and place, i.e. at the hospital or at their home. However, all interviews were conducted in a quiet place in the hospital according to the participant’s wish. The researchers did not know any of the participants, and a tentative date and time for the interview was agreed upon during the phone call. To capture the target population, purposive sampling was chosen as the most appropriate sampling method [12].

### Data collection

During August and September of 2022, the researchers used a semi-structured interview guide to collect data. The guide was developed by the researchers and was translated back and forth between English and Swahili by independent experts to ensure its accuracy. Before its actual use, the guide underwent pilot testing at another hospital other than the study hospitals, and two pilot interviews were carried out to make necessary adjustments, such as modification of unclear questions, to ensure that the informants understood the questions. The guide included open-ended questions about the participants'social demographics, social support, childbirth, pregnancy health, antenatal care, and health providers'attitudes. A reminder phone call was made to each participant before the interview appointment. Written or oral consent was obtained before beginning the interview, and all interviews were conducted in Kiswahili by the research team in a quiet room convenient for the participant and audio recorded with the participant’s permission. The interviews began with opening questions to establish rapport, followed by general questions and ending with a closing session. A research assistant conducted the interviews, with the principal investigator present to ask probing questions and take notes on nonverbal cues. After conducting interviews with 12 participants, nothing new emerged and it was deemed that saturation was met at the tenth interview. To ensure that no new information emerged, two additional interviews were conducted.

### Methodological rigor

When conducting a study, it is crucial to consider factors that ensure its trustworthiness. These factors include transferability, credibility, confirmability and dependability [12, 14]. The study's credibility was guaranteed through the thorough review of all qualitative experts involved. In addition to interview guides, observed gestures and facial expressions were recorded during data collection to ensure triangulation [15]. The interview guide was piloted at another hospital other than the study hospitals; the researchers discussed and made amendments based on the results [14, 16]. The researchers ensured transferability by describing the sampling size, tools, data collection, and processing methods. They provided confirmability by linking findings, conclusions, and interpretations to participant data and including ample quotations to support each theme [14, 15].

### Data processing and analysis

Qualitative content analysis was used to evaluate and interpret the collected data from the interviews. The content analysis aims to find patterns in the data and sort them into themes. The analysis was based on Graneheim and Lundman principles [17]. In step one, the interviews were transcribed verbatim and then read through various times to become familiar with them. In step two, the aims of the study guided the analysis and meaning units; sentences that represented concepts related to the study's objective were chosen from the text. The meaning units were condensed in step three while still conserving their meaning. Step four meant that data collected from the study were subjected to condensation in which meaning units were identified, coded, and subsequently organized into subthemes. During this process, the researchers discussed the naming and meaning of each subtheme. The data were analysed and classified into meaning units, condensed meaning units, codes, and subthemes based on their manifest content and descriptions. In the last part, step five of the analysis, themes were created and discussed thoroughly by all the researchers, representing the underlying meaning and latent content. These themes represent a systematic and rigorous analysis process validated by experts in the field. An example of the analysis process is displayed in Table 1.

**Table 1:** Example of the analysis process following Granheim & Lundman (2004)

**Meaning unit**	**Condensed meaning unit Description close to the text**	**Condensed meaning unit Interpretation of the underlying meaning**	**Subtheme**	**Theme**
Physically, I am affected because my kidneys are not working, they have to undergo dialysis to remove wastes, and I run out of strength due to anaemia.	My kidneys are not working, undergoing dialysis, I run out of strength due to anaemia	Affected the body's physical and mental state due to pregnancy complications.	Physical impact	Living with severe maternal complications
I'm just afraid of eclampsia. I knew I could die and leave my baby behind alone. Who will take care of her?	She is afraid of eclampsia and leave her baby alone	Fear of leaving her baby alone	Mental impact	

### Ethical Approval

Before starting the study, an ethical clearance certificate was obtained from the National Institute for Medical Research in Tanzania (NIMR/HQ/R.8a/Vol. IX/4213) and Kilimanjaro Christian Medical University College (CRERC No 2581). Accordingly, confidentiality was guaranteed during data entry, processing, and storage. The study participants were given codes instead of names for confidentiality. Written or verbal informed consent was obtained from all participants in the case of illiteracy. The participants were made aware that their involvement was voluntary, that they could withdraw at any point without repercussions, and that any data or information about them would be kept confidential. A separate book was used to keep the exact address of the participants, which was used to trace them during the interview or in case of missing appointments and was locked in a place where only the research assistant and PI had access.

## Results

### Demographic characteristics of participants

This study provides a more detailed description of the demographics and obstetric backgrounds of the twelve participants who had experienced severe maternal complications and were admitted to secondary and tertiary hospitals. It sheds light on the diverse experiences of facing maternal difficulties in Kilimanjaro. All participants identified had experienced maternal near misses. Among them, 2 (16.7%) had experienced eclamptic seizures, 3 (25%) had severe preeclampsia, 3 (25%) had severe postpartum haemorrhage (PPH), 2 (16.7%) had acute kidney injury (AKI), 1 (8.3%) had anaemia, and 1 (8.3%) had cardiac disease. The participants'ages ranged from 21 to 41 years. Most of them lived in rural areas, were married, worked in small businesses, were first-time mothers, and had three antenatal visits during their pregnancy, as presented in Table 2.

**Table 2:** Social-demographic characteristics of individual participants (n = 12) *Acute Kidney Injury (AKI)

**Participant**	**Maternal complications**	**Marital status**	**Residential area**	**Occupation**	**Gravidity**	**Parity**	**ANC visits**	**Age**
01	Severe preeclampsia	Not married	Rural	Peasant	1	1	3	24
02	Severe postpartum hemorrhage	Married	Rural	Small business	3	3	4	24
03	Eclampsia	Not married	Rural	Student	1	1	7	23
04	Severe preeclampsia	Cohabiting	Rural	Student	1	1	2	20
05	Severe Postpartum hemorrhage	Cohabiting	Urban	Small business	4	4	3	29
06	Severe postpartum hemorrhage	Married	Rural	Small business	5	4	5	41
07	Cardiac disease	Married	Urban	Small business	3	3	3	25
08	AKI*	Not married	Rural	Small business	1	1	3	21
09	Eclampsia	Married	Rural	Small business	1	1	4	23
10	Severe anemia	Married	Urban	Housewife	1	1	2	21
11	Severe preeclampsia	Cohabiting	Urban	Small business	3	3	3	31
12	AKI*	Married	Urban	Peasant	4	4	1	34

### Results of the content analysis

**Figure 1:**
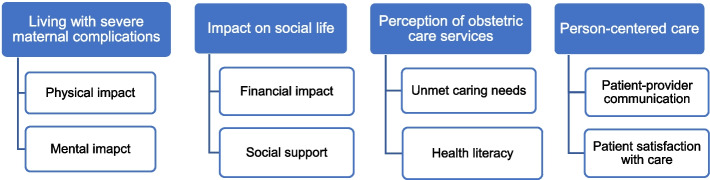
Emerged themes and subthemes

The content analysis yielded four main themes with eight subthemes that represent the lived experience of women who have experienced maternal near misses. The themes that emerged were *living with severe maternal complications, impact on social life, person-centred care,* and *perception of obstetric care services*. Quotations are used to illustrate subthemes, and participant numbers are used to protect confidentiality. The themes and subthemes are presented in Figure 1.

### Theme 1: Living with severe maternal complications

Many women who survive severe pregnancy, childbirth, and postpartum complications reported suffering from physical symptoms due to a change in their body or function of their body and psychological symptoms related to the lived experience of maternal near misses. They felt scared, lonely or ashamed of what they had become due to severe obstetric complications, treatment, and impending risks. Findings related to living with severe maternal complications consisted of two subthemes: *physical impact* and *mental impact*.

#### Physical impact

The lived experience of being near death due to severe maternal complications during pregnancy, childbirth, or postpartum has had physical effects on mothers, such as organ dysfunction, pain, and weakness. The informants were talking about different severe physical problems, for example, kidney failure, requiring dialysis to remove waste, and anaemia causing weakness, which in turn hindered them from caring for their babies as they had planned. It made them dependent on other people or healthcare in a way they were not prepared for Also their female bodies were changed in diverse ways affecting their physical performance. These complications affected them very much and had a great impact on their lives and strength. As the excerpt below highlights:

“Physically, I am affected because my kidneys are not working; they have to undergo dialysis to remove wastes, and I run out of strength due to anaemia”. (P12)

“The difficult birthing experience has damaged my body. The pain is severe, while the private parts have sores, and even the muscles of the legs are aching”. (P6)

#### Mental impact

The analysis revealed that experiencing maternal complications had a significant impact on their mental well-being. The mothers described intense feelings of fear, uncertainty about what had happened uncertainty about the future for themselves and their babies, worry for their health and their baby’s well-being and worry for not recognizing their children who had been transported to the neonatal ward. The mothers still experienced fear when remembering their near-death experience. They feel a lack of support and loneliness during and after the complication. Furthermore, the informants mentioned that they were feeling scared of losing their own or their baby’s life when they were sick, especially before they had been admitted to the ICU. As the excerpt below highlights:

“I feel scared whenever I remember being sick until I was admitted to the ICU”. (P9)

“When I try to feed my child, no milk comes out despite the efforts to suck. It concerns me because I had a difficult birth, and I’m unsure why I’m not producing milk. I’m worried about how this will affect my child’s health”. (P3)

### Theme 2: Impact on Social Life

The findings highlighted how altered social and economic relations impacted continuing life. The altered possibilities meant that social life was restrained from what it used to be, and the economic burden felt heavy, which affected one's overall well-being. The impact of the social life theme resulted from two subthemes, *financial impact and social support.*

#### Financial impact

The financial situations of the informants were severely impacted by the illness and the expenses of being admitted to the hospital. Some informants revealed that they could no longer work, and became dependent on a single family member. They shared that they had to adjust their spending and saving habits, which would have future consequences.

“[….] truly, it is holding back a significant percentage of progress financially due to frequent admissions in recent days. It was costing me a lot”. (P8)

“My life is not the same as it was before. A lot of money has been used. Nevertheless, I can’t do things I used to do, and now it is only my husband we depend on”. (P7)

#### Social support

Social support from close family members and the community is pivotal to maternal well-being and can facilitate recovery. Most participants shared that when they were sick and in the process of recovering, having people who care and offer support was very comforting and gave them a sense of coherence. The excerpt below highlights this:

“It’s comforting to know that you have people who care for and love you even when you are sick; their support helps you in the recovery process”. (P9)

In addition, some women with more severe maternal complications were unable to care for themselves. Sometimes, their family members were allowed to assist them at the healthcare facility. However, some mothers felt deserted by their relatives at home and lacked the necessary support, leading to mental suffering. As highlighted by the excerpt below:

“While I was receiving treatment, my husband was permitted to stay with me at the hospital. We utilized this opportunity to share two to three stories”. (P10)

“I don’t get any support from them. I wash my clothes alone”. (P4)

### Theme 3: Perception of obstetric care services

The findings put forward the importance of being met as a person and receiving care adequate to one’s needs. If this was not done, there was a risk of missed care and adverse events. Additionally, the importance of understanding what health issues meant for one´s recovery was pivotal to a healthy recovery. The two subthemes *unmet caring needs and health literacy* shed light on the importance of the relationship between healthcare providers and patients.

#### Unmet caring needs

When experiencing adverse outcomes and suffering due to ignorance of one's caring needs, puts the mothers and sometimes the unborn baby in a vulnerable position. Sometimes jeopardizing their health. The excerpts below highlight this:

“There is some negligence; healthcare providers have done it. After seeing that the pressure had risen, they were supposed to try to make efforts that would help me lower the pressure. However, I was not given any medicine and told to return home; if my condition changed, come back”. (P3)

“I moaned about the pain until I could not bear it. I cried, and then it was night. Therefore, I followed the nurses there, and they answered that they could not give me anything until the results of the tests were out”. (P8)

Additionally, a shortage of healthcare personnel and a lack of skilled providers can cause extended wait times for services and missed early symptoms of complications, leading to near-miss tragedies.

“You can go at nine in the morning and return at four in the evening. It is a crowded clinic with few healthcare staff”. (P1)

“The reason I decided to change clinics is because the one I was going to was small and lacked experts, as it was a dispensary”. (P7)

#### Health literacy

The well-being of both mothers and newborns depends on participants'access to and understanding of relevant pregnancy health information. In this study, at antenatal visits, most of the informants reported being aware of warning signs to watch out for during pregnancy. However, there was also a discrepancy between being aware and understanding the meaning of severity in the signs one should be aware of.

“During antenatal visits, the midwife talked with me about danger signs; she said if you feel severe headache, you see bloody vaginal discharge, severe heartburn and you do not see clearly, seek treatment immediately”. (P2)

However, few were not aware of pregnancy warning signs. Lack of awareness of warning signs during pregnancy and after giving birth leads to delays in decision-making to seek care promptly

“I do not know those danger signs during pregnancy, and none of the midwives or doctors told me about them”. (P5)

The mothers expressed their feelings about being separated from their babies. They missed them dearly and felt uncertain about how their little ones were faring. The babies were placed in a separate care unit, and often, the mothers were not informed of the reason for the separation. This lack of information about the care provided to their babies increased the mothers'anxiety because they felt that their babies needed them. Additionally, not being able to see their babies due to critical conditions they had was among the most painful experiences of the interviewed women.

As highlighted by the excerpt below:

. “I have given birth to a child; I have not seen him at all, and I don’t know where he is. Therefore, I feel that he has also died”. (P3)

“I asked them, is the baby, okay? They told me you will know there; we are sending you to KCMC. They didn’t tell me anything about the baby”. (P11)

### Theme 4: Person-centered care

The findings in this theme describe how individualized maternal healthcare provided to women based on their needs and preferences created trust in the healthcare providers and satisfaction with the care they received. The theme resulted from two subthemes: patient-provider communication and patient satisfaction with care.

#### Patient-provider communication

Approachable healthcare providers provided a feeling of trust and made the mothers feel safe and grateful. During their interactions with healthcare providers, the mothers exhibited a strong sense of trust in their knowledge and capabilities and felt safe in their care. The providers'responsibility and empathy, as well as their willingness to explain medical issues and provide advice and reassurance, facilitated a good relationship. Excerpts below highlight.

“The staff treated me kindly and conducted some tests. Later that evening, a doctor came and advised me to calm my mind and inquire about my pregnancy’s duration and size, I provided the necessary information after which they recommended an ultrasound test. The doctor then informed me that the baby was in the wrong position, causing discomfort and pain I have”. (P4)

“I speak with all of them very well. There is no problem with the healthcare providers who took care of me here; they always come to check on me and were close to me”. (P6)

#### Patient satisfaction with care

When the informants experienced the care to be good and meaningful, their well-being was improved, which also aided in their recovery and increased patient satisfaction.

“I must say, I received exceptional care at this hospital that has significantly aided my recovery and overall well-being. I feel much better now”. (P8)

“I am satisfied with the service I have received. From the moment I arrived, I have been provided with medicines, undergone investigations, and received exceptional care”. (P9)

## Discussion

Our study explored mothers'lived experiences with severe complications during pregnancy, childbirth and postpartum. We aimed to understand how these women's daily lives were affected by complications within their social and cultural contexts six weeks after delivery. Through their narratives, we found that the impact of these complications was multidimensional and went beyond their hospital stay. According to the findings of the current study, the following themes emerged: living with severe maternal complications, perception of obstetric care services, person-centred care and impact on social life.

### Living with severe maternal complications

The current study showed that women who had severe maternal complications suffer from physical and mental health issues that affect their quality of life [7]. Health issues include weakness, pain after delivery, and organ dysfunction [16, 18], which makes them unable to resume their usual activities [19]. This observation is similar to previous research that has reported a lower quality of life among women who have experienced severe maternal morbidities [16, 20, 21]. Additionally, the present study found that women felt scared and concerned, which harmed their mental well-being. This finding is in line with a study conducted in Iran by TorkmannejadSabzevari et al [11] where it was found that mothers faced multiple problems, failure to adapt to the problem, and numerous physical and psychological issues after an MNM experience. In that study the findings show that the women were afraid of getting pregnant aging, a result that was not present in our study.

### Impact on social life

Our research has discovered that having a strong social support system can lead to a quicker recovery from severe illness, allowing individuals to return to their daily routines. This highlights the importance of social support in improving mental health, reducing stress, and preventing postpartum depression. Research on experiences of MNM conducted in Malaysia and Rwanda [5, 19, 22] support these findings. Additionally, financial difficulties can significantly impact various aspects of life, including mental health and the ability to meet basic needs and delay seeking healthcare services [23]. Our study reveals participants'experiences facing financial constraints due to maternal complications, impacting their lifetime-earning prospects and creating a financial burden for their families. This aligns with a study conducted in Zanzibar on the long-term effects of obstetric near misses [10]

### Perception of obstetric care services

Delaying seeking treatment and maternal deaths are linked to a lack of recognition of pregnancy danger signs [24]. Our research indicates that women were aware of potential danger signs during pregnancy and knew when and where to seek medical assistance. Positive healthcare-seeking behaviour is known to improve maternal and newborn health, and good interaction between healthcare providers and women is crucial in achieving this, which aligns with previous studies conducted in Zanzibar and Ethiopia [24, 25]. Contrary to the systematic review in Ethiopia and a study performed in urban Tanzania, women’s low awareness of potential obstetric danger signs is linked to a lack of exposure to health talk due to low ANC utilization [26, 27]. However, many participants had to wait long at the clinic due to a lack of healthcare staff [28]. This often led to hunger and exhaustion for those who waited longer than expected. This indicates that before attending the antenatal clinic, women must plan with family to provide childcare and cover household activities or take time off from income-generating activities. Failure to make these arrangements may delay or miss antenatal clinic appointments. A study performed in southern Mozambique supports these findings [29]. Furthermore, some women had negative experiences due to healthcare providers neglecting their needs, aligning with previous studies where the neglect of patients'needs and concerns, as well as the humiliation they endure at public healthcare facilities by healthcare providers [11, 22, 30]. Additionally, mothers who experience near misses face communication breakdowns during care, particularly the lack of necessary information about their baby's health. Regular updates about their baby's health status during care can be a simple and cost-effective intervention that can significantly improve their mental well-being.

### Person-centred care

According to our research, healthcare providers offer personalized care that focuses on women's needs and preferences. They achieve this by being approachable, offering advice and reassurance, and establishing trust and relationships. A study conducted in Mtwara also revealed that women appreciated healthcare providers who provided counselling, motivation, and reassurance [31]. Moreover, the study found that most participants reported high satisfaction with hospital care, which aided in their recovery and enhanced the well-being of women who had experienced near-miss events. However, a survey conducted in Nigeria did not support these findings, as women reported dissatisfaction with the antenatal, intrapartum, and postnatal care they received [32].

### Strength and limitations

This study used the Sub-Saharan Africa (SSA) criteria to select maternal near-miss participants. Our study aimed to provide detailed information on the experiences of women who survived severe maternal complications in a vulnerable population identified using the maternal near-miss criteria specific to SSA. Although the number of participants interviewed for this study was small, the findings could provide valuable insights into similar situations. It is essential to consider recall bias, as the women were interviewed six weeks after the event. Nevertheless, earlier interviews were not possible. However, medical records were provided beforehand to aid the interview process and validate the participant’s narrative. Some women could not be interviewed due to staying with relatives living elsewhere (outside the Kilimanjaro Region) until they fully recovered, which could have resulted in selection bias. This means that those interviewed may have had different experiences from those who were missed. Additionally, we did not include in our study the viewpoints of healthcare providers and close family members, which might have added additional perspectives.

## Conclusion

Women who have undergone near-miss obstetric experiences report a mixed range of experiences, encompassing both negative and positive aspects. Maternal near misses have a great impact on women's physical, financial, and mental well-being. These effects may extend beyond the immediate postpartum period. Women also experience long service waiting times, communication barriers, and neglect. Providing practical-, social-, and emotional support during recovery from life-threatening conditions has been highlighted as a crucial aspect of care. Good healthcare, person-centred care, patient education, and psychological support can improve women’s lived experiences during prenatal visits and positive attitudes during and after childbirth. Therefore, comprehensive quality person-centred care, patient education, and support measures should be implemented to enhance trust in the healthcare system and improve the lived experiences of the mothers. Support programs, including psychological support for mothers after hospital discharge, are recommended.

### Availability of data

The data analysed during the current study are not publicly available due to the sensitivity of the research subject and the ethical importance of protecting the anonymity of the study participants.

## Data Availability

The data, analyzed during the current study, are not publicly available due to the sensitivity of the research subject and the ethical importance of protecting the anonymity of the study participants.

## Abbreviations

AKI: Acute Kidney Injury
ANC: Antenatal Clinic
CEmONC: Comprehensive Emergency Obstetric and Newborn Care
ELCT: Evangelical Lutheran Church of Tanzania
GSF: Good Samaritan Foundation of Tanzania
ICU: Intensive Care Unit
KCMC: Kilimanjaro Christian Medical Centre
PPH: Postpartum Hemorrhage
SRQR: Standards for Reporting Qualitative Research
SSA: Sub-Saharan Africa

## References

[1] El-hamid SRA, Amin AE, Yousef HH, El Gelany S. Maternal near miss in EL-Minia Maternity and Children University Hospital: a prospective descriptive study in 2019. 2020;31:1. 10.21608/mjmr.2022.218132.

[2] World Health Organisation (WHO). Maternal Mortality. 2023;1. https://www.who.int/news-room/fact-sheets/detail/maternal-mortality. Accessed 24 Oct 2023.

[3] World Health Organization (WHO). Trends in maternal mortality 2000 to 2020: estimates by WHO, UNICEF, UNFPA, World Bank Group and UNDESA/Population Division. 2023.

[4] World Health Organisation (WHO). Maternal Mortality United Republic of Tanzania 2000-2020 Internationally comparable MMR estimates by the Maternal Mortality Inter-Agency Group (MMEIG): WHO, UNICEF, UNFPA, World Bank Group and the United Nations Population Division. 2023. ISBN 978-92-4-006875-9. https://iris.who.int/bitstream/handle/10665/366225/9789240068759-eng.pdf?sequence=1 Assessed 28 April 2023.

[5] Norhayati MN, Hazlina NHN, Asrenee AR, Sulaiman Z. The experiences of women with maternal near miss and their perception of quality of care in Kelantan, Malaysia: A qualitative study. BMC Pregnancy Childbirth. 2017;17:114. 10.1186/s12884-017-1377-6. 28619038 10.1186/s12884-017-1377-6 PMC5472946

[6] Byrd TE, Ingram LA, Okpara N. Examination of maternal near-miss experiences in the hospital setting among Black women in the United States. Women’s Health. 2022;18. 10.1177/17455057221133830. 10.1177/17455057221133830 PMC9638691 36325622

[7] Abdollahpour S, Heydari A, Ebrahimipour H, Faridhoseini F, Khadivzadeh T. The Mother with Smeary-Death Life : The Lived Experience of Near Miss Mothers. 2021;1–19. 10.21203/rs.3.rs-450126/v1. 10.1186/s12978-021-01321-6 PMC8750850 35012569

[8] Abdollahpour S, Miri HH, Khadivzadeh T. The global prevalence of maternal near miss: A systematic review and meta-analysis. Health Promotion Perspectives. 2019;9:255-62. 10.15171/hpp.2019.35. 31777704 10.15171/hpp.2019.35 PMC6875559

[9] van der Cammen OE, Chobo SP, Kasitu JS, Mwampagatwa I, Mooij R, Hulsbergen MH. Applicability and comparison of the sub-Saharan Africa and original WHO maternal near-miss criteria in a rural hospital in Western Tanzania. Journal of Global Health Reports. 2021;5:1–11. 10.29392/001c.24357.

[10] Herklots T, Yussuf SS, Mbarouk KS, O’Meara M, Carson E, Plug SB, et al. “i lost my happiness, i felt half dead and half alive”-a qualitative study of the long-term aftermath of obstetric near-miss in the urban district of Zanzibar, Tanzania. BMC Pregnancy Childbirth. 2020;20:1–10. 10.1186/s12884-020-03261-8. 10.1186/s12884-020-03261-8 PMC7539452 33028246

[11] TorkmannejadSabzevari M, Eftekhari Yazdi M, Rad M. Lived experiences of women with maternal near miss: a qualitative research. Journal of Maternal and Neonatal Medicine. 2022;35:7158–65. 10.1080/14767058.2021.1945576. 10.1080/14767058.2021.1945576 34219597

[12] Polit, D.F. and Beck, C.T. Nursing Research: Generating and Assessing Evidence for Nursing Practice. 10th Edition, Wolters Kluwer Health, Philadelphia. 2017. 10.1016/j.iccn.2015.01.005.

[13] O’Brien BC, Harris IB, Beckman TJ, Reed DA, Cook DA. Standards for Reporting Qualitative Research. Acad Med. 2014;89:1245–51. 10.1097/acm.0000000000000388. 24979285 10.1097/ACM.0000000000000388

[14] Elo S, Kääriäinen M, Kanste O, Pölkki T, Utriainen K, Kyngäs H. Qualitative Content Analysis SAGE Open. 2014;4:1-10. 10.1177/2158244014522633.

[15] Maher C, Hadfield M, Hutchings M, de Eyto A. Ensuring Rigor in Qualitative Data Analysis: A Design Research Approach to Coding Combining NVivo With Traditional Material Methods. International Journal of Qualitative Methods. 2018;17:1–13. 10.1177/1609406918786362.

[16] Amegavluie REA, Ani-Amponsah M, Naab F. Women’s experiences of surviving severe obstetric complications: a qualitative inquiry in southern Ghana. BMC Pregnancy Childbirth. 2022;22:1–9. 10.1186/s12884-022-04538-w. 35296276 10.1186/s12884-022-04538-w PMC8928636

[17] Graneheim UH, Lundman B. Qualitative content analysis in nursing research: Concepts, procedures and measures to achieve trustworthiness. Nurse Education Today. 2004;24:105–12. 10.1016/j.nedt.2003.10.001. 10.1016/j.nedt.2003.10.001 14769454

[18] Kaye DK, Kakaire O, Nakimuli A, Osinde MO, Mbalinda SN, Kakande N. Lived experiences of women who developed uterine rupture following severe obstructed labor in Mulago hospital, Uganda. Reproductive Health. 2014;11:1–9. 10.1186/1742-4755-11-31. 24758354 10.1186/1742-4755-11-31 PMC3997795

[19] Semasaka JPS, Krantz G, Nzayirambaho M, Munyanshongore C, Edvardsson K, Mogren I. “Not taken seriously”-A qualitative interview study of postpartum Rwandan women who have experienced pregnancy-related complications. PLoS One. 2019;14:1–17. 10.1371/journal.pone.0212001. 10.1371/journal.pone.0212001 PMC6373944 30759136

[20] Machiyama K, Hirose A, Cresswell JA, Barreix M, Chou D, Kostanjsek N, et al. Consequences of maternal morbidity on health-related functioning: A systematic scoping review. BMJ Open. 2017;7. 10.1136/bmjopen-2016-013903. 10.1136/bmjopen-2016-013903 PMC5719332 28667198

[21] Angelini CR, Pacagnella RC, Parpinelli MA, Silveira C, Andreucci CB, Ferreira EC, et al. Quality of life after an episode of severe maternal morbidity: Evidence from a cohort study in Brazil. Biomed Research International. 2018;9348647. 10.1155/2018/9348647. 10.1155/2018/9348647 PMC6076926 30105265

[22] Bagambe PG, Umubyeyi A, Nyirazinyoye L, Luginaah I. Women’s experiences and perceptions on the impacts of maternal near miss and related complications in Rwanda: A qualitative study. African Journal of Reproductive Health. 2022;26:63–71. 10.29063/ajrh2022/v26i5.7. 37585098 10.29063/ajrh2022/v26i5.7

[23] Påfs J, Musafili A, Binder-Finnema P, Klingberg-Allvin M, Rulisa S, Essén B. Beyond the numbers of maternal near-miss in Rwanda - a qualitative study on women’s perspectives on access and experiences of care in early and late stage of pregnancy. BMC Pregnancy Childbirth. 2016;16:1–11. 10.1186/s12884-016-1051-4. 27590589 10.1186/s12884-016-1051-4 PMC5010768

[24] Bakar RR, Mmbaga BT, Nielsen BB, Manongi RN. Awareness of danger signs during pregnancy and post-delivery period among women of reproductive age in unguja island, zanzibar: A qualitative study. African Journal of Reproductive Health. 2019;23:27–36. 10.29063/ajrh2019/v23i1.3. 31034169 10.29063/ajrh2019/v23i1.3

[25] Mesele TT, Syuom AT, Molla EA. Knowledge of danger signs in pregnancy and their associated factors among pregnant women in Hosanna Town, Hadiya Zone, southern Ethiopia. Front Reprod Heal. 2023;5:1–8. 10.3389/frph.2023.1097727. 10.3389/frph.2023.1097727 PMC10036572 36970710

[26] Geleto A, Chojenta C, Musa A, Loxton D. WOMEN ’ s Knowledge of Obstetric Danger signs in Ethiopia (WOMEN ’ s KODE): a systematic review and meta-analysis. Systematic Reviews. 2019;8:1–15. 10.1186/s13643-019-0979-7. 10.1186/s13643-019-0979-7 PMC6388496 30803443

[27] Mwilike B, Nalwadda G, Kagawa M, Malima K, Mselle L, Horiuchi S. Knowledge of danger signs during pregnancy and subsequent healthcare seeking actions among women in Urban Tanzania : a cross-sectional study. BMC Pregnancy Childbirth. 2018;18:1–8. 10.1186/s12884-017-1628-6. 10.1186/s12884-017-1628-6 PMC5751870 29295710

[28] Biza A, Jille-Traas I, Colomar M, Belizan M, Requejo Harris J, Crahay B, et al. Challenges and opportunities for implementing evidence-based antenatal care in Mozambique: A qualitative study. BMC Pregnancy Childbirth. 2015;15:1–10. 10.1186/s12884-015-0625-x. 26330022 10.1186/s12884-015-0625-x PMC4557743

[29] Gong E, Dula J, Alberto C, De Albuquerque A, Steenland M, Fernandes Q, et al. Client experiences with antenatal care waiting times in southern Mozambique. BMC Health Serv Res. 2019;6:1–9. 10.1186/s12913-019-4369-6. 10.1186/s12913-019-4369-6 PMC6670125 31370854

[30] Kwame A, Petrucka PM. Communication in nurse-patient interaction in healthcare settings in sub-Saharan Africa: A scoping review. Int J Africa Nurs Sci. 2020;12:100198. 10.1016/j.ijans.2020.100198.

[31] Kwezi HA, Mselle LT, Leshabari S, Hanson C, Pembe AB. How communication can help women who experience a maternal near-miss: A qualitative study from Tanzania. BMJ Open. 2021;11:1–8. 10.1136/bmjopen-2020-045514. 10.1136/bmjopen-2020-045514 PMC8562531 34725070

[32] Okonofua F, Ogu R, Agholor K, Okike O, Abdus-Salam R, Gana M, et al. Qualitative assessment of women’s satisfaction with maternal health care in referral hospitals in Nigeria. Reproductive Health. 2017;14:1–8. 10.1186/s12978-017-0305-6. 28302182 10.1186/s12978-017-0305-6 PMC5356406

